# Genetic characterization of melon accessions in the U.S. National Plant Germplasm System and construction of a melon core collection

**DOI:** 10.1186/s43897-021-00014-9

**Published:** 2021-09-07

**Authors:** Xin Wang, Kaori Ando, Shan Wu, Umesh K. Reddy, Prabin Tamang, Kan Bao, Sue A. Hammar, Rebecca Grumet, James D. McCreight, Zhangjun Fei

**Affiliations:** 1 Boyce Thompson Institute, Cornell University, Ithaca, NY 14853 USA; 2 U.S. Department of Agriculture-Agricultural Research Service, Crop Improvement and Protection Research Unit, Salinas, CA 93905 USA; 3 Nunhems USA, Inc, Acampo, CA 95220 USA; 4 Gus R. Douglass Institute and Department of Biology, West Virginia State University, Institute, WV 25112 USA; 5 U.S. Department of Agriculture-Agricultural Research Service, Natural Products Utilization Research Unit, Thad Cochran Research Center, P.O. Box 1848, Oxford, MS 38677 USA; 6 Department of Horticulture, Michigan State University, East Lansing, MI 48824 USA; 7 U.S. Department of Agriculture-Agricultural Research Service, Robert W. Holley Center for Agriculture and Health, Ithaca, NY 14853 USA

**Keywords:** Melon, *C. melo*, Germplasm, Genetic characterization, Core collection, Genome-wide association study

## Abstract

Melon (*C. melo* L.) is an economically important vegetable crop cultivated worldwide. The melon collection in the U.S. National Plant Germplasm System (NPGS) is a valuable resource to conserve natural genetic diversity and provide novel traits for melon breeding. Here we use the genotyping-by-sequencing (GBS) technology to characterize 2083 melon accessions in the NPGS collected from major melon production areas as well as regions where primitive melons exist. Population structure and genetic diversity analyses suggested that *C. melo ssp. melo* was firstly introduced from the centers of origin, Indian and Pakistan, to Central and West Asia, and then brought to Europe and Americas. *C. melo ssp. melo* from East Asia was likely derived from *C. melo ssp. agrestis* in India and Pakistan and displayed a distinct genetic background compared to the rest of ssp. *melo* accessions from other geographic regions. We developed a core collection of 383 accessions capturing more than 98% of genetic variation in the germplasm, providing a publicly accessible collection for future research and genomics-assisted breeding of melon. Thirty-five morphological characters investigated in the core collection indicated high variability of these characters across accessions in the collection. Genome-wide association studies using the core collection panel identified potentially associated genome regions related to fruit quality and other horticultural traits. This study provides insights into melon origin and domestication, and the constructed core collection and identified genome loci potentially associated with important traits provide valuable resources for future melon research and breeding.

**Supplementary Information:**

The online version contains supplementary material available at 10.1186/s43897-021-00014-9.

## Core

Genetic characterization of 2083 melon accessions in the U.S. National Plant Germplasm System provides insights into melon genetic diversity, origin and domestication, and facilitates the construction of a melon core collection that captures the majority of the genetic variation in the germplasm. Genome-wide association studies identified potentially associated genome regions related to fruit quality and other horticultural traits, providing valuable information for melon research and breeding.

## Background

Melon (*C. melo* L.; 2n = 2 × =24) is a highly polymorphic member of the Cucurbitaceae family. It is an economically important fruit crop grown primarily in temperate and semi-tropical regions with a worldwide production of 27.5 million metric tonnes in 2019 (FAOSTAT; http://www.fao.org/faostat/en). Melon fruits can be eaten fresh as a vegetable or a dessert, cooked, pickled, or dried (McCreight et al. 2013), while its seeds are an important source of vegetable oil for human consumption and for nutraceutical applications (Jacks et al. 1972; McCreight et al. 2013; Mallek-Ayadi et al. 2019).

Although it is clear that melon was domesticated about 4000 years ago (Pitrat et al. 2000; Pitrat 2017), its center of origin has been controversial (Kerje and Grum 2000; Luan et al. 2008; Sebastian et al. 2010; Endl et al. 2018). Africa was regarded for many years as the center of origin of melon because of the existence of the large number of wild *Cucumis* species (Whitaker and Davis 1962; Robinson and Decker-Walters 1997). Later it was concluded that Asia is the center of origin, and the abundant diversity of melon across India and East Asia further supports this conclusion (Akashi et al. 2002; Dhillon et al. 2007; Tanaka et al. 2007; Dwivedi et al. 2010). However, a recent analysis of a diverse collection of *C. melo* accessions from Asia, Australia and African, suggested two independent melon domestication events, one in Africa and the other in Asia (Endl et al. 2018). Furthermore, recent studies based on the large-scale genome resequencing data demonstrated three independent domestication events in melon, two in India and one in Africa (Zhao et al. 2019), consistent with the abundant diversity of melon found across India and Africa (Akashi et al. 2002).

Melon classification has been confusing scientists, farmers, regulators, gardeners and consumers for more than 150 years. Two subspecies were recognized in 1980 based on a single ovary difference: *agrestis* and *melo* (Jeffrey 1980), and two subsequent revisions of melon typified prior tendencies to increase the number of groups or condense them (Pitrat et al. 2000). Much of the tremendous variability in melon is considered to be human-induced and maintained, and should, therefore, be treated as horticultural variation (Kirkbride Jr. 1993) that is subject to categorization according to the nomenclature for cultivated plants (Brickell et al. 2009). Pitrat et al. (2000) reviewed the various melon classification schemes that were often based on incomplete samplings of melons, and organized them into 16 botanical *varietas* in two subspecies, *agrestis* (5 *varietas*) and *melo* (11 *varietas*). This scheme was updated with usage of horticultural groups (Burger et al. 2010). Later Pitrat (2017) abolished ssp. *melo* and *agrestis*, and identified 19 horticultural groups based on a combination of characters. However, a subsequent analysis of melon nuclear ribosomal internal transcribed spacer (ITS) regions aligned with geographical origin thereby provided an objective basis for designation of two subspecies: *melo*, found in Asia and Australia, and *meloides*, found in Africa (Endl et al. 2018). A recent analysis based on genome-wide single nucleotide polymorphisms (SNPs) similarly found geographical distinctions between African and Indian domestications (Zhao et al. 2019). Furthermore, separate selection sweeps and allele differences for key domestication traits such as fruit size, flesh thickness, acidity, and loss of bitterness were observed between the two Indian groups, spp. *agrestis* and spp. *melo*, indicating the occurrence of two domestication processes in southeast Asia (Zhao et al. 2019).

The U.S. National Plant Germplasm System (NPGS) has one of the largest collections of melon germplasm with more than 2000 available accessions, including cultivars, breeding lines, landraces, and genetic stocks collected from more than 70 countries. A majority of the accessions are designated only to the species level, e.g., *C. melo*, though many have subspecies and/or *varietas* designations. As few are correctly identified for horticultural groups, regardless of the classification scheme, genetic analysis of the collection can help us to understand the diversity present in the collection and relationships among accessions. Advances in next generation sequencing technologies have contributed to the development of new cost-effective genotyping platforms, such as the genotyping-by-sequence (GBS) approach. The relatively large number of SNPs derived from GBS can be used for genome-wide association studies (GWAS) and genetic diversity analyses (Huang and Han 2014; Gur et al. 2017). Genome-wide SNPs can also aid the selection of a core collection to capture the maximum genetic diversity with minimal redundancy. In this study, we genotyped 2083 melon accessions in the NPGS using the GBS approach. We first corrected potential misclassifications for some melon accessions recorded in the NPGS by extensively reviewing the associated data (e.g., place of origin, photos) for all accessions combined with knowledge from literature. We then performed population structure, phylogenetic relationship, pattern of linkage disequilibrium (LD), and nucleotide diversity analyses for different melon groups using the genotyping data, which were further used to select a core collection of 383 accessions comprising the majority of genetic diversity in the germplasm population. Thirty-five morphological characters were evaluated in the core collection, and GWAS were then performed to identify potential loci in the melon genome that determine several important quantitative and qualitative traits.

## Results

### Genotyping of the NPGS melon collection

**Fig. 1:**
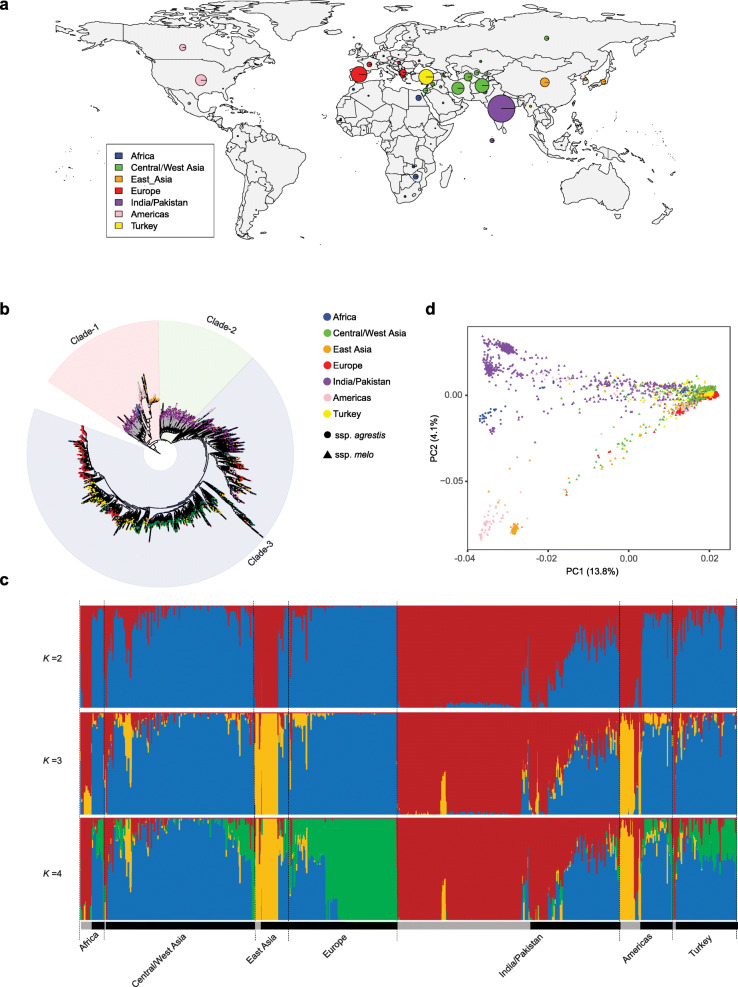
Geographic distribution, phylogeny and population structure of melon accessions. **a** Geographical distribution of the 2083 melon accessions in the U.S. National Plant Germplasm System (NPGS). The circle size indicates the relative sampling number from each region. **b** Rooted maximum-likelihood phylogenetic tree of the 2083 melon accessions. Gray and black branch colors indicate the ssp. *agrestis* and the ssp. *melo*, respectively. Three cucumber accessions whose sequence data were downloaded from NCBI SRA (SRR543204, SRR543227 and SRR543228) were used as the outgroup. **c** Model-based clustering with *K* from 2 to 5. The colors and length of vertical bars represent different ancestral populations and the relative contributions in each of the 2083 melon accessions. The gray and black bars indicate ssp. *agrestis* and ssp. *melo*, respectively. **d** Principal component analysis (PCA) of the 2083 melon accessions with PC1 and PC2 explaining 13.8 and 4.1% of variance, respectively

The melon germplasm collection maintained in the U.S. NPGS contained 2083 accessions, of which 1850 were classified into *C. melo ssp. melo* and 160 into *C. melo ssp. agrestis*, while 73 were not classified into either subspecies (Supplementary Table 1). However, according to the classification proposed by Pitrat et al. (2000), 1269 were classified into *C. melo ssp. melo*, 451 into *C. melo ssp. agrestis*, and 363 unclassified. To obtain a more accurate classification of the NPGS melon accessions, we manually reviewed the data provided in the NPGS for all melon accessions, including their places of origin and photos, combined with literature (e.g., Pitrat et al. 2000) and our knowledge of the materials. Finally, 1546 were classified into *C. melo ssp. melo*, 535 into *C. melo ssp. agrestis*, and two remained unclassified (Supplementary Table 1). Based on their geographic distributions, the 2083 melon accessions were divided into seven groups, 75 from Africa, 478 from Central/West Asia, 113 East Asia, 339 from Europe, 708 from India/Pakistan, 166 from Americas and 204 from Turkey (Fig. 1a; Supplementary Table 1). Most of the *C. melo ssp. agrestis* accessions (422 out of 527) were from India/Pakistan and the rest were from Americas (59) and other regions (46).

We genotyped the 2083 NPGS melon accessions using the GBS technology. A total of ~ 2.6 billion reads were generated, from which 347,056 unique tags with at least 10 read counts, corresponding to a total of ~ 1.6 billion reads, were obtained and used for SNP calling. About 70.1% of these reads were aligned to unique positions while 8.4% were aligned to multiple positions in the melon reference genome (v3.5.1; Garcia-Mas et al. 2012). Approximately 2.4% (7.9 Mb) of the genome was covered by the GBS reads, which is commonly observed for the GBS data (Elshire et al. 2011). A total of 89,204 raw SNPs were identified, with an average of one SNP per 4.56 kb, of which 78,125 were biallelic. After filtering SNPs with MAF ≤0.01 and missing data rate > 50%, a total of 27,471 biallelic SNPs were obtained, with an average of one SNP per 14.8 kb (Supplementary Table 2), and these SNPs were used in the subsequent analyses unless otherwise indicated.

### Phylogenetic relationships and population structure of *C. melo* accessions

To infer phylogenetic relationships among the 2083 melon accessions, a maximum likelihood (ML) tree was constructed with the identified SNPs, using three cucumber accessions as the outgroup. Nine accessions from Ghana, Senegal, Zimbabwe and Zambia, including two wild African melons (PI 505611 and PI 482393) identified in a previous study (Zhao et al. 2019), were positioned close to the outgroup, suggesting that the primitive *C. melo ssp. agrestis* from Africa might be the most closely related to the direct progenitor of cultivated melon. Three major clades were identified in the remaining accessions, which were mainly comprised of two ssp. *agrestis* subgroups and one clade of ssp. *melo* accessions (Fig. 1b and Supplementary Figure 1). Subspecies *agrestis* accessions were, for the most part and regardless of origin, clustered in the deep clades, intermixed with some ssp. *melo* accessions from India/Pakistan and East Asia (Fig. 1b and Supplementary Figure 1). The East Asia ssp. *melo* and ssp. *agrestis* accessions and Americas ssp. *agrestis* accessions were located in one of the ssp. *agrestis* clades, and they might be derived from India/Pakistan ssp. *agrestis* melons. The third clade contained all the ssp. *melo* accessions from India/Pakistan, Americas, Central/West Asia, Europe, and Africa (Fig. 1b). Consistent with India/Pakistan as the centers of origin for cultivated melon (Mallick and Masui 1986), most of the ssp. *melo* accessions from India/Pakistan were in the deep branches in the ssp. *melo* clade and distant from accessions from other groups. We identified a small subclade that mainly consisted of ssp. *melo* accessions from Europe and Americas, with some accessions from Africa, India/Pakistan and Central/West Asia in the deeper branch (Fig. 1b). The ssp. *melo* accessions from Turkey were clearly separated into two clusters, with one closely related to Central/West Asia accessions and the other closely related to Europe accessions. The few ssp. *agrestis* accessions that were intermixed in the ssp. *melo* clade including those from India/Pakistan could be mis-classified or reflect outcrossing events (Supplementary Figure 1).

African melons collected from two geographic regions, ssp. *agrestis* from southern Africa and ssp. *melo* from northern Africa, were mainly located in the clade 1 and clade 3, respectively. The southern African accessions in the clade 1 were separated into two groups (Fig. 1b). Besides the nine accessions positioned closed to the outgroup, 24 accessions from Zimbabwe and Zambia were located with the India/Pakistan ssp. *agrestis* accessions, suggesting that they could have been brought from the India/Pakistan region to southern Africa by migrants (Zhao et al. 2019).

Population structure of the melon accessions was then investigated. At *K* = 2, the ssp. *agrestis* accessions had one ancestral background while admixture was present in ssp. *melo* accessions (Fig. 1c). At *K* = 3, the East Asia and Americas ssp. *agrestis* accessions were separated from the India/Pakistan ssp. *agrestis* accessions. At *K* = 4, a new subgroup emerged within ssp. *melo*, with the European accessions having a distinct background and the rest ssp. *melo* accessions displaying mixed genetic backgrounds shared with both ssp. *melo* and *agrestis*. Principal component analysis (PCA) revealed a similar pattern of their phylogenetic relationships and population structure (Fig. 1d). India/Pakistan ssp. *agrestis* accessions formed a centralized distribution and were distant from the India/Pakistan ssp. *melo* accessions, which displayed a discrete distribution. Consistent with the phylogeny, Americas ssp. *agrestis* and East Asia accessions each formed a cluster, and the ssp. *melo* outside India/Pakistan were clustered together. These results suggested that the East Asia ssp. *agrestis* and *melo* accessions and Americas ssp. *agrestis* could be directly domesticated from India/Pakistan ssp. *agrestis*, while the remaining ssp. *melo* accessions were derived from India/Pakistan ssp. *melo*.

### Linkage disequilibrium decay, nucleotide diversity and population divergence

**Fig. 2:**
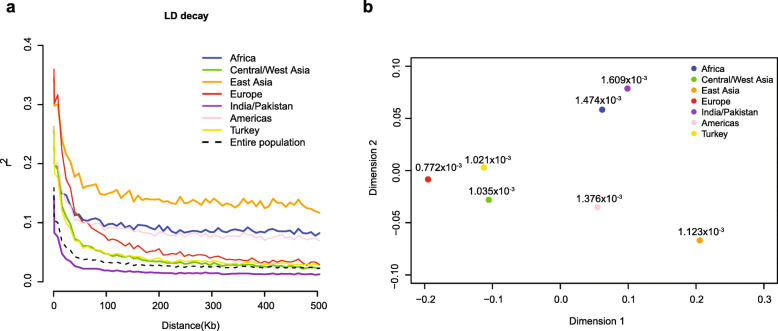
Linkage disequilibrium (LD) of melon and population divergence across different melon groups. **a** Estimated average LD decay of melon in different groups. **b** Multidimensional scaling of pairwise *F*_ST_ values between different melon groups. The number near each dot indicates the nucleotide diversity (π) within the corresponding group

The LD, measured as the correlation coefficient (*r*^2^) between two SNPs, was calculated for each group. The LD decay was measured as the physical distance at which LD decayed to half of its maximum value. The LD decay was around 16 kb (*r*^2^ = 0.067; maximum *r*^2^ = 0.159) for the entire population (Fig. 2a). When the seven groups were analyzed independently, the LD decay varied dramatically. The East Asia group had the longest LD decay distance, 56 kb, while the India/Pakistan group had the shortest, 8 kb. The remaining groups showed comparable LD decay patterns and physical distances (24 kb, 32 kb, 24 kb, 16 kb, and 16 kb for Europe, Africa, Americas, Central/West Asia and Turkey, respectively) (Fig. 2a).

The nucleotide diversity was estimated for each group by the average value of genome-wide nucleotide diversity (π) (Fig. 2b). The nucleotide diversity for Europe, Americas, Middle East, Central/West Asia, and East Asia groups ranged from 0.772 × 10^− 3^ to 1.123 × 10^− 3^, which were much lower than that for India/Pakistan (1.609 × 10^− 3^) and Africa (1.473 × 10^− 3^) groups. The high π values for India/Pakistan and Africa groups were consistent with the hypothesis that melon originated from these two regions, and revealed that cultivated melon accessions in these two groups contain greater genetic diversity than in the other groups.

Population divergences among the seven groups were estimated using pairwise fixation index (*F*_ST_) values. Pairwise weighted *F*_ST_ values among Americas, Central/West Asia, Africa, India/Pakistan, Turkey ranged from 0.049 to 0.305. The values between East Asia and the other six groups except Europe ranged from 0.151 to 0.326, while the values between Europe and other six groups except East Asia ranged from 0.116 to 0.305 (Supplementary Table 3). Visualization of pairwise weighted *F*_ST_ values using multiple dimensional scaling (MDS) revealed a clear distinction between the East Asia group and other groups (Fig. 2b). The maximum *F*_ST_ value was 0.407, which occurred between the Europe group and the East Asia group, indicating that accessions in these two groups could be domesticated and improved independently, and retained distinctly different pools of genetic diversity in response to adaptation to their respective ranges of environment and consumption demand, e.g., culinary uses, organoleptic preferences. The π, LD and *F*_ST_ of the Europe group suggested that accessions from this group have undergone a more severe population bottleneck during domestication compared with the East Asia group.

### Development of the core melon germplasm collection

**Fig. 3:**
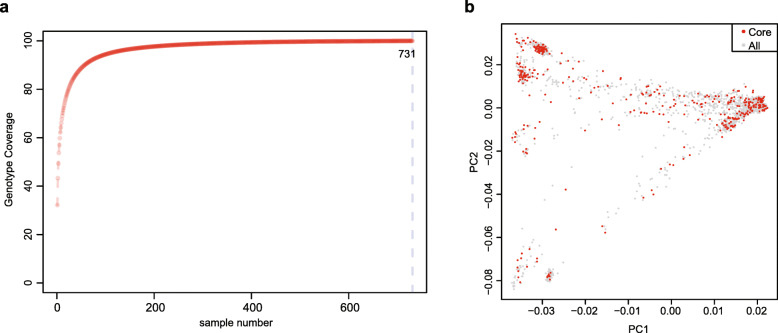
Development and evaluation of the melon core collection. **a** Coverage of allelic diversity versus number of selected accessions. The dash line indicates the minimal number of samples (731) covering all the allelic diversity. **b** Principal component analysis (PCA) of melon accessions. Red dots represent the accessions in the core collection; gray dots represent the accessions not in the core collection

Establishment of a core collection provides a subset of representative accessions that captures the majority of the allelic diversity in the entire collection and can be used for QTL mapping, GWAS, marker development, gene cloning and crop breeding. Our analysis indicated that the 731 top-ranked accessions could capture 100% of the allelic diversity of the entire melon germplasm collection in the U.S. NPGS (Fig. 3a). A core set of 383 accessions were selected from the 2083 melon accessions, capturing 98.96% of the entire allelic diversity in the NPGS melon germplasm collection. The core collection included accessions that had historical importance, including source of important disease resistance traits, e.g., PI 414723 (McCreight et al. 1984; McCreight et al. 1992), or representatives of horticultural groups, e.g., ‘Charentais’. The top two groups with the highest proportions in the 383-accession core collection were from Africa (21; 28%) and India/Pakistan (183; 25.8%), which displayed abundant genetic variations. The remaining groups displayed comparable proportion, with 70 (14.6%) from Central/West Asia, 17 (15.0%) from East Asia, 31 (18.7%) from Americas, 32 (9.4%) from Europe, and 29 (14.2%) from Turkey (Supplementary Table 4). The accessions in the core collection were classified into 11 groups based on the three major phylogenetic clades and their geographical origins within each clade (Fig. 1). The African and Americas accessions were found in clades 1 and 3 (14 Africa accessions in clade 1 and 7 in clade 3; 13 Americas accessions in clade 1 and 18 in clade 3). Most East Asia accessions belonged to clade 1 (14 accessions) and most accessions from Central/West Asia (67 accessions), Europe (31) and Turkey (28) were in clade 3. The India/Pakistan accessions were found in all three clades (58 in clade 1, 71 in clade 2 and 54 in clade 3) (Supplementary Table 4). PCA analysis of the accessions in the melon core collection exhibited a pattern nearly identical to that of the accessions in the entire collection (Fig. 3b), further supporting the broad representation of the core accessions over the NPGS melon germplasm collection.

### Phenotypic variation in the melon core collection

The melon core collection of 383 accessions was used to evaluate the morphological variability of melon from a wide range of geographic origins. Plants were grown under the same environment and evaluated for 35 horticultural traits including fruit morphology and sweetness, inflorescence characters and disease resistance (Supplementary Table 5).

**Fig. 4:**
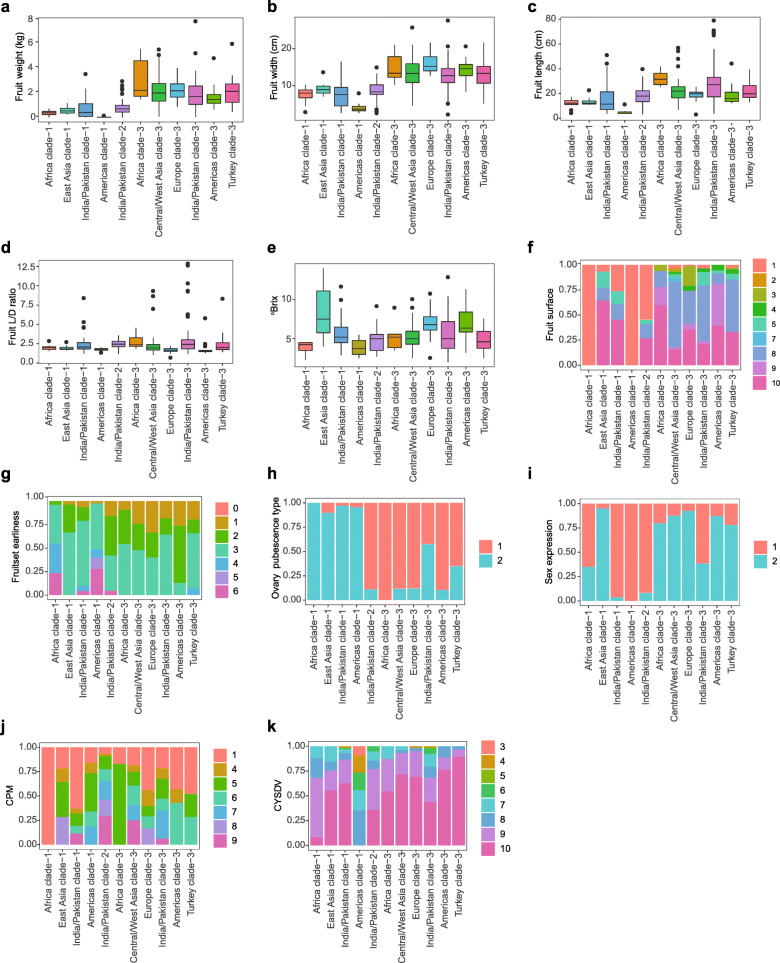
Phenotypic variation in the melon core collection. Distribution for eleven horticultural (**a**-**i**) and disease resistance (**j**-**k**) traits. **a** Mature fruit weight (kg). **b** Mature fruit width (cm). **c** Mature fruit length (cm). **d** Mature fruit length/width (L/D) ratio. **e** Soluble solids content (^o^Brix). **f** Fruit surface type. 1, Smooth; 2, Grainy; 3, Finely wrinkled; 4, Deeply wrinkled; 5, Shallowly wavy; 6, Rare warts; 7, Numerous warts; 8, Lightly corked/netted; 9, Heavily corked/netted; 10, Vein tracts. **g** Fruit set earliness. 0, Very early; 1, Early; 2, Intermediate; 3, Late; 4, No female flowers at anthesis; 5, No male flowers at anthesis; 6, No flowers present. **h** Ovary pubescence type. 1, Spreading hairs (ssp. *melo*); 2, Appressed hairs (ssp. *agrestis*). **i** Sex expression. 1, Monoecious (male and female on same plant); 2, Andromonoecious (male/female and male on sample plant). **j** Resistance to cucurbit powdery mildew (CPM). 1, No evidence of disease; 4, Few colonies present, sporulation; 5, Scattered colonies, sporulation; 6, Numerous colonies, sporulation; 7, ~ 50% of adaxial surface covered with hyphae and spores, few colonies on abaxial surface, abundant sporulation; 8, > 50% of adaxial surface covered with hyphae and spores, scattered colonies on abaxial surface; abundant sporulation; petiole and internodes may be infected; 9, > 75% of adaxial surface covered with hyphae and spores, numerous or coalesced colonies on abaxial surface; heavy sporulation; petiole and internodes usually infected. **k** Resistance to *Cucurbit yellow stunting disorder virus* (CYSDV). 3, 20–30% foliage symptomatic; 4, 30–40% foliage symptomatic; 5, 40–50% foliage symptomatic; 6, 50–60% foliage symptomatic; 7, 60–70% foliage symptomatic; 8, 70–80% foliage symptomatic; 9, 80–90% foliage symptomatic; 10, > 90% foliage symptomatic. Trait descriptors are also provided in Supplementary note

The evaluated fruit quantitative and qualitative traits included fruit weight, fruit length, fruit width, fruit length/width radio, soluble solid content, fruit surface, and fruit set earliness. Higher fruit weight values as well as variability were observed in clade 3 accessions than in those from clades 1 and 2 regardless of geographic origins (Fig. 4a). High variabilities for fruit length, fruit width and fruit length/width radio were seen in the India/Pakistan group in clade 3, whereas the lowest morphological variabilities were found in Americas and Africa groups in clade 1 (Fig. 4b-d). The accessions in East Asia of clade 1, and Europe and Americas of clade 3 exhibited the highest fruit soluble solid content, while the values for the remaining groups were similar (Fig. 4e). Observations related to the fruit surface revealed that Africa and Americas accessions in clade 1 were generally characterized by a smooth surface, and the other groups exhibited a wide variability in the fruit surface characteristics (Fig. 4f). On the other hand, the Africa and Americas groups in clade 1 had higher variability in fruit set earliness than the other groups (Fig. 4g).

Traits related to inflorescence characters included sex expression and the type of ovary pubescence that distinguishes ssp. *agrestis* and ssp. *melo*. As expected, appressed hairs on the ovary were the dominant phenotype for accessions in clade 1, which was mostly composed of ssp. *agrestis* accessions, and spreading hairs were observed in groups containing ssp. *melo* accessions (Fig. 4h). Most groups had one significant dominant sex type, with more than 75% of the accessions exhibiting either monoecious or andromonoecious, except for Africa accessions in clade 1 and India/Pakistan accessions in clade 3, which were a mixture for both types (Fig. 4i).

The resistances to cucurbit powdery mildew (CPM) incited by *Podosphaera xanthii* and *Cucurbit yellow stunting disorder virus* (CYSDV) showed different patterns among groups (Fig. 4j-k). Africa accessions in clade 1 exhibited high resistance to CPM and the percent of accessions with high susceptibility were found higher in the Central/West Asia accessions in clade 3 and India/Pakistan accessions in clade 2 than that in other groups. Africa accessions in clade 3 exhibited low variability of CPM resistance, with 80% of accessions exhibiting intermediate resistance and 20% exhibiting high resistance. The remaining groups showed intermediate susceptibility to CPM (Fig. 4j). Different from the pattern of CPM, most groups exhibited a high susceptibility expect Americas accessions in clade 1, of which most accessions exhibited a higher or intermediate resistance (Fig. 4k).

### Genome-wide association studies using the core collection

**Fig. 5:**
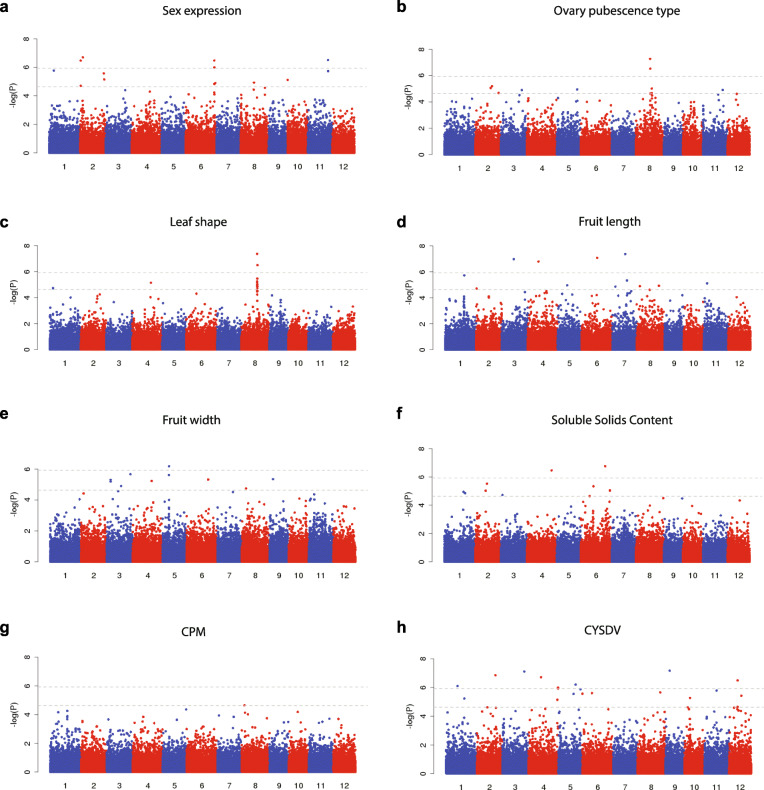
Manhattan plot of genome-wide association studies (GWAS) of interesting traits. **a** Sex expression. **b** Ovary pubescence type. **c** Leaf shape. **d** Fruit length. **e** Fruit width. **f** Soluble solids content. **g** Cucurbit powdery mildew (CPM). **h** Cucurbit yellow stunting disorder virus (CYSDV). In each plot, the horizontal dashed lines correspond to the Bonferroni-corrected significance thresholds at α = 0.05 (top) and α = 1 (bottom), respectively

GWAS is a rapid method to identify quantitative trait loci (QTLs) and potential candidate genes for important agronomic traits and has been widely applied in various crops (Huang and Han 2014). In this study, GWAS were performed using the 383 core collections for a number of important traits, including sex expression, ovary pubescence, leaf and fruit morphology, and fruit flesh soluble solid content (Supplementary Table 6). Fifteen SNPs on chromosome 2, 6 and 11 were found to be associated with sex expression (Fig. 5a), with the most significant SNP (chr2: 1,805,185) located near the *CmACS-7* gene (*MELO3C015444*) known to determine sex expression in melon (Boualem et al. 2008; Gur et al. 2017). Five SNPs spanning 10.5–14.6 Mb on chromosome 8 and seven other SNPs distributed on chromosomes 2, 3, 5 and 11 were associated with ovary pubescence, a trait that could distinguish ssp. *melo* and ssp. *agrestis* (Fig. 5b). However, a previous reported GWAS signal for ovary pubescence was not identified in our study, probably due to the populations used for GWAS with different structures (Zhao et al. 2019). A narrow region on chromosomes 8 (~ 14.60–14.66 Mb) harboring 15 associated SNPs was associated with leaf shape (Fig. 5c). Thirteen SNPs on chromosome 3, 4, 6, 7 were associated with fruit length (Fig. 5d), among which two (chr1: 23926443 and chr8:1858632) overlapped with previous known QTLs controlling fruit weight (Perpina et al. 2016; Pereira et al. 2018). Ten SNPs were detected to be associated with fruit width (Fig. 5e), among which four were located in or near the QTLs known to control fruit size and weight (Pereira et al. 2018). Eleven SNPs on chromosome 1, 2, 3, 4 and 6 were detected to be associated with soluble solid content of fruit flesh (Fig. 5f**)**, and the five SNPs on chromosome 6 were located in the previous identified QTLs *fruqsc6.2*, *sscqsc6.4* and *fruqsc6.6* (Argyris et al. 2017)**.** One SNP on chromosome 8 was found to be associated with CPM resistance (Fig. 5g). Twenty SNPs were associated with the CYSDV resistance, among which eight were significantly associated with this trait (Fig. 5h). One SNP on chromosome 5 (chr5: 20827177) was located near a known QTL of CYSDV resistance (Tamang et al. 2021).

## Discussion

The genetic composition of 2083 available melon accessions maintained in the NPGS, collected from ssp. *agrestis* and ssp. *melo*, were characterized using the GBS technology, providing 89,204 raw SNPs and 78,152 biallelic SNPs. The revealed phylogenetic relationships and population structure using these SNPs were consistent with our current understanding about melon (Zhao et al. 2019). Accessions from India/Pakistan were found in the deepest branches of both the ssp. *agrestis* and ssp. *melo* clades, supporting two independent domestication events in the India/Pakistan region (Zhao et al. 2019). The genetic diversity was highest in the accessions from the domestication origins, India/Pakistan and Africa, and lower in groups from other geographic regions, which was in agreement with the previously discovered bottleneck during melon domestication (Zhao et al. 2019). East Asia ssp. *melo* and ssp. *melo* from other geographic origins had a distant phylogenic relationship and, thereby, distinct genetic backgrounds. East Asia ssp. *melo* shared a genetic background with ssp. *agrestis* and could be derived from India/Pakistan ssp. *agrestis*. These results suggested that ssp. *melo* from East Asia and ssp. *melo* from other geographic regions were domesticated independently. The Central/West Asia ssp. *melo* accessions were positioned closely to the ssp. *melo* from India/Pakistan in the phylogenetic tree, suggesting that ssp. *melo* might have been introduced from India/Pakistan to Central/West Asia first, and then spread to the Europe and Americas. Turkey ssp. *melo* accessions were found in two subclades, most clustered with European accessions and a few mixed in the Central/West Asia group, reflecting the different ssp. *melo* types cultivated in that country, which straddles Europe and Asia and through which the ancient Silk Road connected Asia (Central/West Asia and India/Pakistan) with Europe. The genetic characterization of the 2083 melons in the U.S. NPGS provided initial knowledge for the evolutionary and dispersal of melon in different geographic regions. A main objective of characterizing genetic variation of this large-scale melon samples was to develop a core collection to facilitate breeding. A valuable core collection of 383 accessions was determined, representing most of the genetic variation and capturing the phenotypic diversity in melon. This core collection panel provides valuable materials and information for future melon genetic research and breeding. GWAS analysis using the core collection identified associated SNPs in the known QTL regions underlying traits such as sex expression, fruit size and quality and disease resistance. In addition, potential novel associated SNPs were identified for ovary pubescence, leaf and fruit morphology, and fruit quality, yet the limited number of SNP makers identified using the GBS technology restricted the discovery of target genes in the QTL regions. Deep sequencing of the core collection can reveal more genetic variation needed for future GWAS and QTL-seq analyses to uncover the genetic basis of horticultural traits of melon.

## Methods

### Sample collection and DNA extraction

Seeds of NPGS melon accessions (Supplementary Table 1) were planted in successive plantings in a greenhouse in plastic, multi-cell flats filled with commercial potting mix (Premium Growers Mix Sunland Garden Products, Watsonville, CA) with cells sized 6 cm × 4 cm × 6 cm deep, two seeds per cell, and six cells per line. Seedlings were watered with dilute (1:100) 20 N-20P-20 K fertilizer.

About 100 mg fresh leaf tissue was collected from a young seedling representing each melon accession. The leaf tissue was freeze-dried and then ground to a fine powder using 5/32″ stainless steel balls (AbbottBall, West Hartford, CT) in a Retsch Mixer Mill (Retsch, Newtown, PA). DNA was isolated using the Plant DNA DS Kit (M1130; Omega Bio-Tek, Norcross, GA). The DNA was quantified with the Quant-iT PicoGreen dsDNA Kit (Invitrogen, Carlsbad, CA), and its quality was assessed by electrophoresis of undigested and *Hind*III-digested DNA on agarose gels.

### GBS analysis and SNP calling

Genotyping of the melon accessions was performed following the GBS protocol described in Elshire et al. (2011), using the *ApeK*I restriction enzyme (NEB, Beverly, MA). GBS libraries were sequenced on a HiSeq 2500 system with read length of 101 bp. The TASSEL 5.0 GBS discovery pipeline (Glaubitz et al. 2014) was used for SNP calling. Briefly, raw GBS sequencing reads that possessed a barcode and a restriction enzyme cut site were identified using GBSSeqToTagDBPlugin in TASSEL with parameters ‘-kmerLength 90 -minKmerL 30 -mnQS 10 -c 10 -maKmerNum 200000000’. Tags were retrieved and reformatted using TagExportToFastqPlugin, and those supported by at least ten reads were kept and then mapped to the melon reference genome sequence (v3.5.1) (Garcia-Mas et al. 2012) using BWA v0.7.13 (Li and Durbin 2009) with default parameters. Based on the alignments, positions of aligned tags were determined using SAMtoGBSdbPlugin, and SNPs were identified from the aligned tags using DiscoverySNPCallerPluginV2 with default parameters. SNPs were filtered based on their missing data rate and minor allele frequencies (MAF) using VCFtools v 0.1.15 (Danecek et al. 2011).

### Phylogenetic and population genomic analyses

Biallelic SNPs with MAF ≥ 1% and missing rate ≤ 50% were used for phylogenetic and population structure analyses. A maximum likelihood tree was constructed with IQTREE v1.6.8 (Nguyen et al. 2015), with three cucumber accessions used as the outgroup. The ggtree package v1.10.5 (Yu et al. 2017) was used to visualize and annotate the phylogenetic tree. PCA was performed using Plink v1.9 (Purcell et al. 2007). Population structure analysis was performed using FastSTRUCTURE (Raj et al. 2014) for each *K* with *K* = 2–4.

LD decay for all pairs of SNPs within 500 kb were calculated using PopLDdecay v3.27 (Zhang et al. 2019) with the following parameters: -MaxDist 500 -MAF 0.05 -Het 0.88 -Miss 0.999. The unfiltered raw SNPs were used to calculate the nucleotide diversity (π) and population fixation index (*F*_ST_) using VCFtools v 0.1.15 (Danecek et al. 2011). The average nucleotide diversity for each group was calculated as the sum of nucleotide diversity divided by the number of bases covered by GBS reads in the melon reference genome. The pairwise weighted *F*_ST_ values were calculated and transformed into two-dimensional values using the cmdscale function in R.

### Construction of the core collection

A subset of the NPGS melon accessions were selected based on the GBS SNP data using GenoCore (Jeong et al. 2017) with the following parameters: ‘-d 0.01% -cv 100%’, to capture the majority of the allelic diversity in the germplasm. Additional accessions were selected based on their historical importance, e.g., source of important disease resistance traits or representation of a horticultural group.

### Phenotyping of the core collection

Accessions in the core collection were evaluated for 35 vegetative, flower and fruit characters in a non-replicated field planting at the University of California, Desert Research and Education Center, Holtville, CA; 33 of the traits were evaluated using scales of The International Plant Genetic Resources Institute (IPGRI) descriptors for melon and many are included in the IPGRI subset of highly discriminating melon descriptors (IPGRI 2003) (Supplementary note). Seeds were planted on March 7, 2018 in dry soil and irrigated via subsurface (ca. 20 cm depth) drip lines in raised, flat beds on 2 m centers, and grown using cultural practices common for melon production in the desert southwest United States. Each experimental plot was ca. 4.0 m long and consisted of five hills spaced ca. 61 cm apart, two seeds per hill, followed by a ca. 1.5 m buffer to provide space for better separation of plots.

### Genome-wide association studies

A total of 78,152 raw biallelic SNPs were used to construct the Balding-Nichols kinship (K) matrix (Balding and Nichols 1995). The missing genotypes were imputed using the k-nearest neighbor algorithm implemented in fillGenotype (Huang et al. 2010). In order to determine the optimal parameters that contribute to a high imputation accuracy and filling rate, four accessions (PI 357792, PI 614364, PI 512514 and PI 406737) with lowest missing and heterozygous SNP rates were selected, and 10, 20 and 30% SNP sites were randomly masked as missing and imputed using fillGenotype with the following parameter combinations: w (50, 65, 80), p (− 3, − 5, − 7), k (3, 5, 7), and r (0.7, 0.75, 0.8). The optimal combination of parameters (w = 30, k = 9, *p* = − 9, r = 0.8) were used to impute the raw biallelic SNPs after comparing the filling rate and accuracy rate of each combination of parameters. After imputation, only biallelic SNPs with MAF ≥ 1% and missing data rate ≤ 50% (a total of 43,000 SNPs) were used for GWAS analysis. GWAS were performed using the linear mixed model (LMM) implemented in the EMMAX software (Kang et al. 2010). The genome-wide significance thresholds of the GWAS were determined using the Bonferroni correction at α = 1 and α = 0.05 for suggestive and significant associations, respectively, as described in Li et al. (2012), which corresponded to raw *P* values of 2.33 × 10^− 5^ [−log10(P) of 4.63] and 1.16 × 10^− 6^ [−log10(P) of 5.93] in this study.

### Supplementary Information

**Additional file 1:** **Supplementary Figure 1.** The maximum-likelihood phylogenetic tree of the 2083 melon accessions annotated by ssp. *agrestis* (blue) and ssp. *melo* (orange).

**Additional file 2 **: **Supplementary Table 1.** List of melon accessions in the U.S. National Plant Germplasm System (NPGS). **Table 2.** Summary statistics of the identified SNPs for each chromosome. **Table 3.** Pairwise *F*_ST_ values between different melon groups. **Table 4.** Numbers of accessions from different groups in the melon core collection. **Table 5.** Phenotype data collected from the 383 accessions in the core collection. **Table 6.** SNPs associated with agronomic traits identified through GWAS.

## Data Availability

Raw and filtered SNPs in VCF format are available at the Cucurbit Genomics Database (http://cucurbitgenomics.org; Zheng et al. 2019).
