# Supplementary material for: Genetic characterization of melon accessions in the U.S. National Plant Germplasm System and construction of a melon core collection
Source: Mol Hortic. 2021 Sep 7;1:11. doi: 10.1186/s43897-021-00014-9 (PMC10515074; doi:10.1186/s43897-021-00014-9)
Supplement: Supplementary file 1 — Additional file 1: Supplementary Figure 1. The maximum-likelihood phylogenetic tree of the 2083 melon accessions annotated by ssp. agrestis (blue) and ssp. melo (orange). [file 43897_2021_14_MOESM1_ESM.pdf]

Supplementary Note

Melon phenotype descriptors

A total of 35 melon vegetative, flower and fruit characters were evaluated for accessions in the melon core collection. Fruit characters were evaluated at the maturity stage unless otherwise indicated. Most phenotype descriptors were adopted from The International Plant Genetic Resources Institute (IPGRI) "Descriptors for melon *Cucumis melo* L.": **https://www.bioversityinternational.org/fileadmin/_migrated/uploads/tx_news/Descriptors_for_melon__Cucumis_melo_L.__906.pdf**

Descriptors of melon phenotypes investigated in this study

**Phenotype**	**Descriptors**
Fruit shape	1 Globular (round); 2 Flattened; 3 Oblate; 4 Elliptical; 5 Pyriform (pear-like); 6 Ovate; 7 Acorn; 8 Elongate; 9 Scallop (like a scallop shell); 99 Other
Predominant fruit skin color	1 White; 2 Light-yellow; 3 Cream; 4 Pale green; 5 Green; 6 Dark green; 7 Blackish-green; 8 Orange; 9 Brown; 10 Grey; 99 Other
Secondary fruit skin color	1 White; 2 Light-yellow; 3 Cream; 4 Pale green; 5 Green; 6 Dark green; 7 Blackish-green; 7 Orange; 8 Brown; 9 Grey; 99 Other
Secondary skin color pattern	0 No secondary skin colour; 1 Speckled (spots <0.5 cm); 2 Spotted, blotchy (spots >0.5 cm); 3 Striped (bands that run from peduncle to blossom scar); 4 Short streaked (elongated marks that are continuous; from one end the other and <4 cm in length; 5 Long streaked (as 4 but >4 cm); 99 Other
Internal skin color	1 White; 2 Green; 3 Yellow; 4 Orange (yellow-red); 5 Salmon (pink-red); 99 Other
Main color of flesh	1 White; 2 Yellow; 3 Cream; 4 Pale green; 5 Green; 6 Pale orange; 6 Orange (yellow-red); 7 Salmon (pink-red); 99 Other
Flesh color of outer layer	1 White; 2 Yellow; 3 Cream; 4 Light green; 5 Green; 6 Orange (yellow-red); 7 Salmon (pink-red); 99 Other
Placenta color	1 White; 2 Green; 3 Yellow; 4 Orange (yellow-red); 5 Salmon (pink-red); 99 Other
No. Placenta	1 3; 2 5; 99 some other number, commonly 4 or 6
Slip	1 slips; 2 does not slip
Fruit surface	1 Smooth; 2 Grainy; 3 Finely wrinkled; 4 Deeply wrinkled; 5 Shallowly wavy; 6 Rare warts; 7 Numerous warts; 8 Lightly corked/netted; 9 Heavily corked/netted; 10 Vein tracts; 99 Other
Bitterness	(n=10) 3 Low; 5 Intermediate; 7 High
Plant size	3 Small (<1 m3); 5 Intermediate (1-3 m3); 7 Large (>3 m3)
Canopy cover	3 Open (internodes completely exposed to sunlight); 5 Intermediate; 7 Closed (internodes completely covered by foliage)
Canopy depth	Distance from ground to top of canopy. 3 <10 cm; 5 10-20 cm; 7 >20 cm
Leaf color	1 Light green; 1.1 yellow-green; 1.2 grey-green; 2 Green; 3 Dark green; 4 Variable
Leaf petiole hairiness	1 Sparsely hispid; 2 Hispid; 3 Hispidulous; 4 Retrorse strigose; 5 Lanate (rarely); 99 Other "nearly glabrous"
Petiole hair stiffness	1 stiff; 2 soft
Ovary hair	1 Short (< 1 mm); 2 Intermediate (1-5 mm); 3 Long (> 5 mm)

Ovary hairiness	0 None; 1 Velvety; 2 Medium; 3 Hairy
Ovary shape	1 Flat; 2 Round; 2.5 Oval; 2.6 Pyriform 3 Long; 4 Very long
Glabrous leaf	1 glabrous; 2 hirsute, a.k.a. hairy
Sepal size	1 Small (normal); 2 Leaf like
Ovary pubescence type	1 Spreading hairs (ssp. *melo*); 2 Appressed hairs (ssp. *agrestis*)
Fruit set earliness	Based on presence of flowers and fruit on May 15, 2018, ca. 10-weeks post-planting. 0 Very early; 1 Early; 2 Intermediate; 3 Late; 4 No female flowers at anthesis; 5 No male flowers at anthesis; 6 No flowers present
Sex expression	1 Monoecious (male and female on same plant); 2 Andromonoecious (male/female and male on sample plant); 3 Gynoecious (female); 4 Male sterile; 5 Female sterile; 99 Other
Leaf shape	1 Entire; 2 Trilobate; 3 Pentalobate; 4 3-palmately lobed; 5 5-palmately lobed
Plant growth habit	1 Compact (shortened internode length between 0.5 and 2.5 cm, bush habit); 2 Dwarf (internode length 4-6 cm, short in height, rarely exceeds 1 m); 3 Determinate (vining habit in which ends of branches terminate in cluster of flowers or leaves, such plants stop growing during growing season; 4 Indeterminate (vining habit, in which branches continue to grow throughout growing season); 5 Multilateral (many branches); 99 Other
CPM (cucurbit powdery mildew)	1 No evidence of disease; 2 Trace of hyphae, no detectable sporulation; 3 Hyphae restricted, no detectable sporulation; 4 Few colonies present, sporulation; 5 Scattered colonies, sporulation; 6 Numerous colonies, sporulation; 7 ~50% of adaxial surface covered with hyphae and spores, few colonies on abaxial surface, abundant sporulation; 8 >50% of adaxial surface covered with hyphae and spores, scattered colonies on abaxial surface; abundant sporulation; petiole and internodes may be infected; 9 >75% of adaxial surface covered with hyphae and spores, numerous or coalesced colonies on abaxial surface; heavy sporulation; petiole and internodes usually infected
CYSDV (cucurbit yellow stunting disorder virus)	1 <10% foliage symptomatic; 2 10-20% foliage symptomatic; 3 20-30% foliage symptomatic; 4 30-40% foliage symptomatic; 5 40-50% foliage symptomatic; 6 50-60% foliage symptomatic; 7 60-70% foliage symptomatic; 8 70-80% foliage symptomatic; 9 80-90% foliage symptomatic; 10 >90% foliage symptomatic

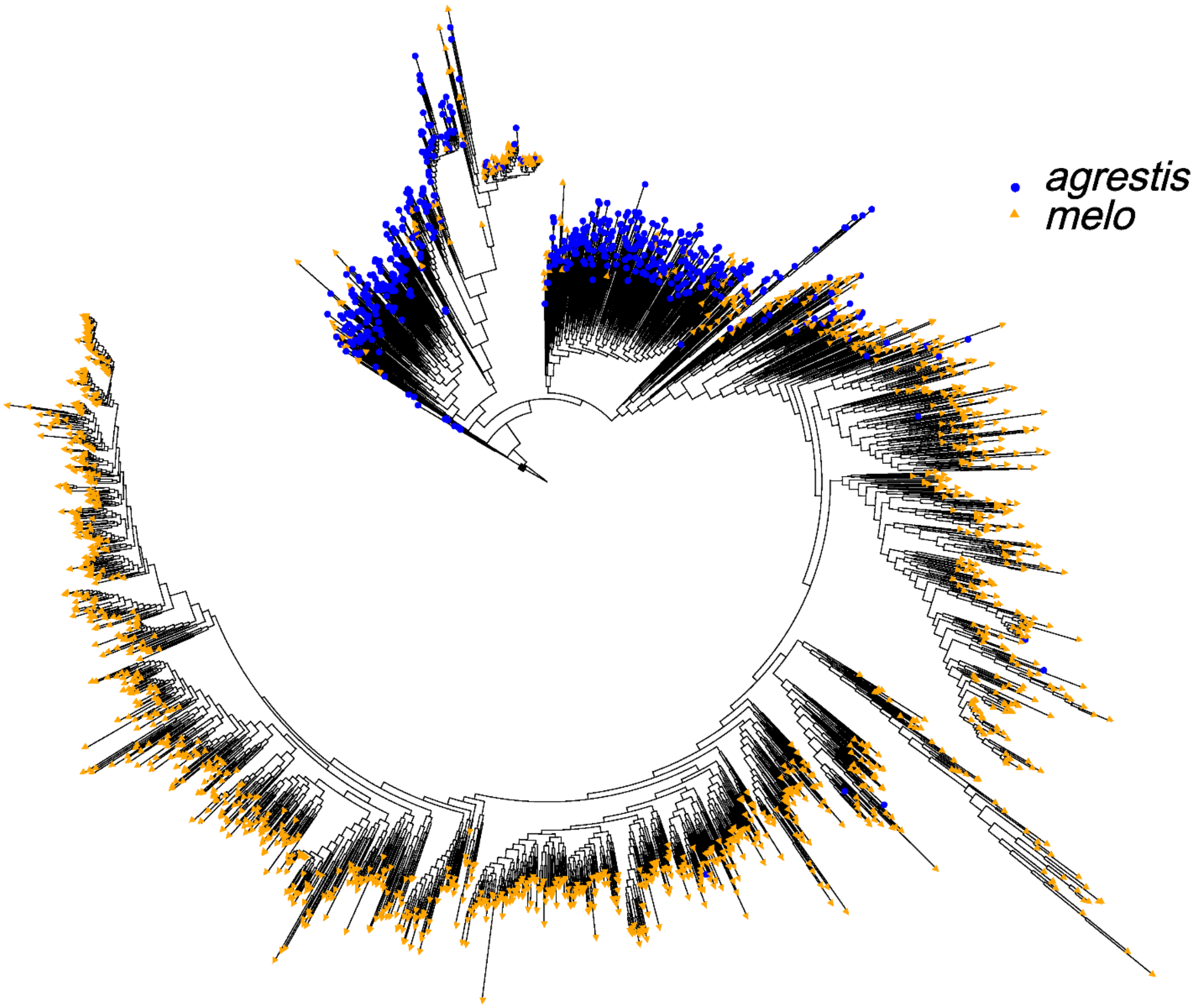

Supplementary Figure 1 The maximum-likelihood phylogenetic tree of the 2,083 melon accessions annotated by ssp. *agrestis* (blue) and ssp. *melo* (orange).
